# Finger millet (*Eleusine coracana* L.): from staple to superfood—a comprehensive review on nutritional, bioactive, industrial, and climate resilience potential

**DOI:** 10.1007/s00425-024-04502-2

**Published:** 2024-08-17

**Authors:** Simardeep Kaur, Arti Kumari, Karishma Seem, Gurkanwal Kaur, Deepesh Kumar, Surbhi Verma, Naseeb Singh, Amit Kumar, Manish Kumar, Sandeep Jaiswal, Rakesh Bhardwaj, Binay Kumar Singh, Amritbir Riar

**Affiliations:** 1 ICAR-Research Complex for North Eastern Hill Region, Umiam, Meghalaya 793103 India; 2 Bihar Agricultural University, Sabour, Bhagalpur, 813210 India; 3 ICAR-Indian Agricultural Research Institute, New Delhi, 110012 India; 4 Punjab Agricultural University, Ludhiana, Punjab 141004 India; 5 ICAR-National Institute of Plant Biotechnology, New Delhi, 110012 India; 6 College of Agriculture and Applied Sciences, Utah State University, Logan, UT 84322 USA; 7 ICAR-National Bureau of Plant Genetic Resources, New Delhi, 110012 India; 8 Department of International Cooperation, Research Institute of Organic Agriculture, FiBL, 11 Frick, Switzerland

**Keywords:** Finger millet, Nutritional profile, Biological activities, Industrial potential, Climate resilience, Crop improvement, Stress tolerance traits

## Abstract

**Main conclusion:**

This review discusses the Finger millet's rich nutritional profile, bioactive potential, and industrial applications, combined with its climate resilience, which make it a promising crop for enhancing food security and promoting sustainable agriculture. This review also highlights its significant potential to address malnutrition and mitigate climate change impacts.

**Abstract:**

The emergence of Finger millet from “poor man’s staple food” to “a nutrient rich cereal” has encouraged the need to explore this crop at a wider scale. It is a highly significant crop due to its rich nutritional and bioactive profile, diverse biological activities, and promising industrial applications, along with the high climate resilience. This comprehensive review evaluates its nutritional composition by comparing favorably with other cereals and millets and emphasizing its potential to address malnutrition and enhance food security. Furthermore, it explores the phytochemical/bioactive potential and strategies to enhance their bioavailability followed biological activities of Finger millet by highlighting its various health-promoting properties. The review also discusses industrial potential of finger millet including its role in nutraceutical and functional food production, as well as bioenergy generation. In addition, role of Finger millet as a climate-resilient crop; specifically, the available genetic resources and identification of genes and quantitative trait loci (QTLs) associated with major stress tolerance traits have also been discussed. By providing a comprehensive synthesis of existing knowledge, this study offers valuable insights for researchers, policymakers, and stakeholders engaged in efforts to promote sustainable agriculture, enhance food and nutrition security, and mitigate the impacts of climate change.

## Introduction

The growing concern of the population related to nutrient-sufficient diets and the vulnerability of agriculture productivity to unpredictable climate requires diversity in crop production. In this scenario, millets, one of the oldest foods known to humans, often overlooked in the approbation of major staple crops including wheat and rice under the influence of urbanization and industrialization could be a game-changer due to their exceptional qualities, such as climate resilience and minimal input demand compounded by their rich nutritional profile. Due to the growing interest of the population in healthier grains, the world’s demand for millets is expected to reach $12 billion by 2025 (Kumari et al. 2023). At present, global millet production totals 30.73 million tons, with India contributing 11.42 million tons (37%), securing its position as the leading producer worldwide. Despite this, globally, there is an estimated decline of ~ 25.71% in the area under millet cultivation from 1961 to 2018 (Meena et al. 2021).

**Fig. 1:**
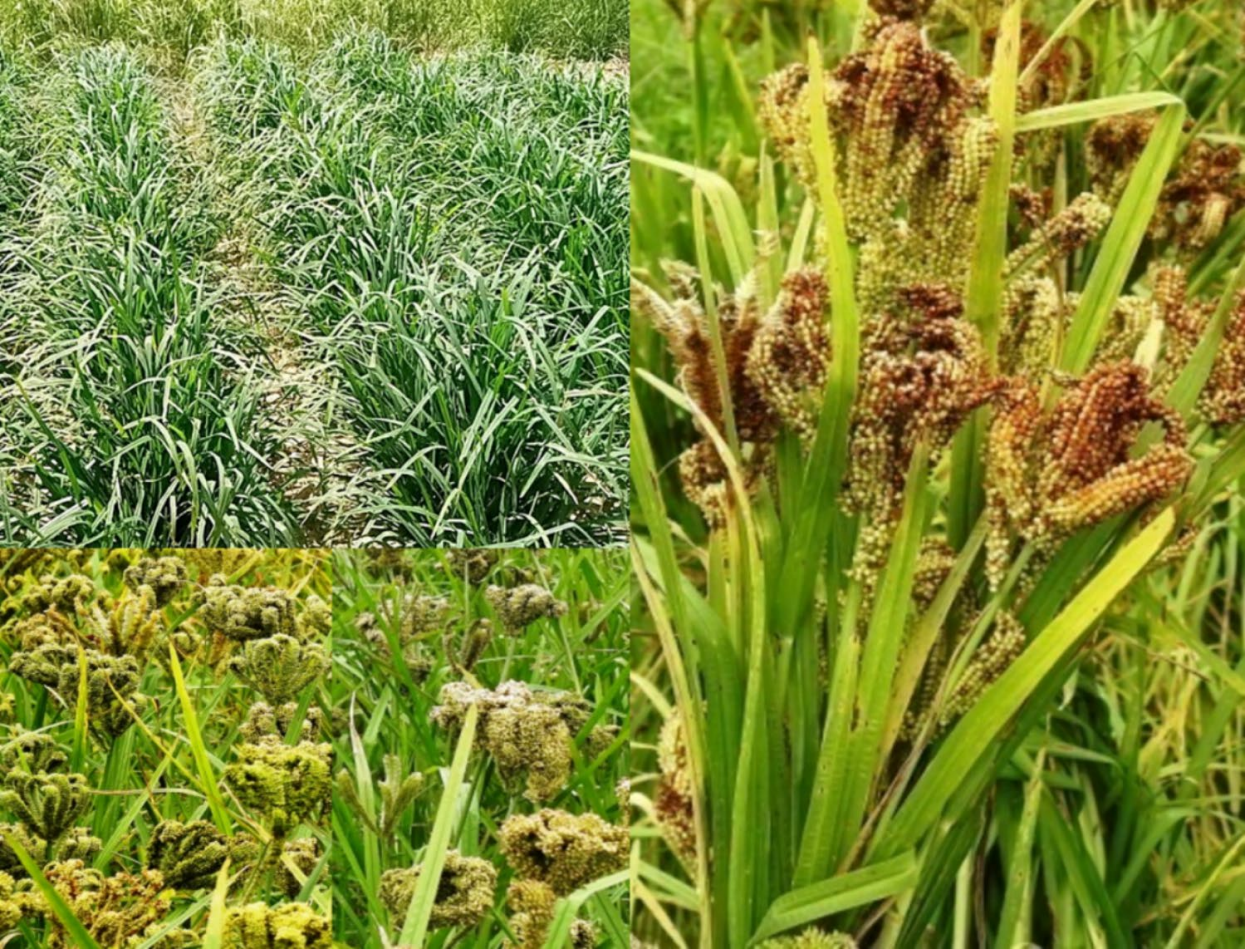
Finger millet in field

Among the six major small-grained cereal crops, namely, Finger millet (*Eleusine coracana*), Foxtail millet (*Setaria italica*), Kodo millet (*Paspalum scrobiculatum*), Proso millet (*Panicum miliaceum*), Barnyard millet (*Echinochloa* spp.), and Little millet (*Panicum sumatrense*) (Si and CG 2016). The foremost, a minor millet originating from Africa accounts for 85% of total production in India (Divya et al. 2013). It is a C4 crop, commonly known as Mandua and Ragi in India (Fig. 1). Finger millet is native to the Ethiopian highlands and commonly cultivated in more than 25 countries including Uganda, Nepal, India, Sri Lanka, Bangladesh, East China, Tanzania, Kenya, etc. (Rathore et al. 2019). The generic name *Eleusine* originated from the Greek goddess of cereals, “*Eleusine*”, while the common name Finger millet implies the panicle’s “Finger-like branching” (Gupta et al. 2017). This is an allopolyploid with 2*n* = 4*x* = 36 chromosomes, developed from a cross between two diploid species, *E. indica* (AA) and *E. floccifolia* or *E. tristachya* (BB) (Hilu and Wet 1976). As a gluten-free option, it offers an additional advantage for individuals with stomach-related issues and brings forth a plethora of health advantages, including anti-diabetic effects (for type 2 diabetes mellitus), anti-diarrheal properties, antiulcer properties, anti-inflammatory benefits, antitumorigenic effects (for K562 chronic myeloid leukemia), atherosclerogenic effects, antimicrobial properties, and antioxidant properties (Chandrasekara and Shahidi 2010; Devi et al. 2014). Most of these wellness advantages are due to the dietary fiber fraction and the polyphenols of which 85% are benzoic acid derivatives and the remaining are cinnamic acid derivatives and flavonoids (Chethan and Malleshi 2007).

Furthermore, to cope with the formidable climate challenges, Finger millet can be considered as a key candidate in enhancing climate-smart agriculture and diversifying food baskets for humans. This species has been reported to perform well under extreme climatic conditions (Hilu and Wet 1976). The industrial demand for Finger millets is also on the rise due to the potential to be processed into nutritious value-added products. This review discusses the multifaceted aspects of Finger millet, including its nutritional benefits, phytochemical and bioactive composition, processing method for their enhanced bioavailability, biological characteristics, industrial and nutraceutical applications, and its potential to contribute to climate resilience for sustainable food and nutrition security. Targeted towards researchers, policymakers, agriculturalists, and nutritionists, this review offers a comprehensive understanding of the diverse benefits and potentials of Finger millet, providing valuable insights for addressing global challenges related to food security and nutrition.

## Nutritional composition

The Finger millet kernel consists of three main components: the outermost testa layer (seed coat), the intermediate endosperm layer, and the innermost embryo. Finger millet distinguishes itself from other millets such as pear millet, foxtail millet, proso millet, and kodo millet due to its five-layered testa. This distinguishing feature may account for the advantage of Finger millet’s higher dietary fibre content. Finger millet is rich source of essential nutrients as stated in Table 1. It comprises approximately 65–75% carbohydrates, 5–8% protein, 1–2% fat, 15–20% dietary fibre, and 2.5–3.5% minerals (Devi et al. 2014; Chandra et al. 2016). Finger millet has a total energy content of 300–350 kcal. It is rich in phytochemicals, polyphenols, flavonoids, and tannins, which makes it useful in foods and nutraceuticals (Chandrasekara and Shahidi 2012a; Chandra et al. 2016).

**Table 1:** Nutritional composition of Finger millet

Category	Content* (%)	Content** (%)	Content*** (%)	Subcategory	Content* (%, g/100 g or mg/100 g)	Content** (%, g/100 g or mg/100 g)	Content*** (%, g/100 g or mg/100 g)	References*
Macronutrients								
Carbohydrates	65–75%	72.6%	66.82%	Starch	60%	**–**	62.13 g	Bhatt et al. (2003) and Devi et al. (2014)
				Amylose	15–20%	16%	**–**	
				Amylopectin	80–85%	**–**	**–**	
				Cellulose	–	**–**	**–**	
				Lignins	1.4–1.8%	**–**	**–**	
				Free sugars	0.04–0.6%	**–**	34 g	
				NSP	1–2%	**–**	**–**	
Dietary fiber	18.6%	–	11.18%	Crude fibre	3.5 g	3.6 g	–	Ramulu and Udayasekhara Rao (1997), Rathore et al. (2019) and Kalsi and Bhasin (2023)
				Insoluble dietary fiber	12%	–	9.51 g	
				Soluble dietary fiber	2%	–	1.67 g	
				Hemicellulose A	1.40%	**–**	**–**	
				Hemicellulose B	1.90%	**–**	**–**	
Protein	5–8%	7.7%	7.16%	Prolamin	35–50%	24.6–36.2%	**–**	Gull et al. (2014)
				Globulins and albumins	8–15%	17.3–27.6%	**–**	
Amino acids	–	–	–	Valine	–	5.0 g	5.65 g	Amadou et al. (2013)
				Leucine	10.8 g	7.0 g	8.86 g	
				Isoleucine	4.3 g	4.0 g	3.70 g	
				Lysine	2.2 g	5.5 g	2.83 g	
				Cysteine	–	3.5g^a^	1.48 g	
				Methionine	2.9 g	–	2.74 g	
				Arginine	3.4 g	–	–	
				Tryptophan	–	–	0.19 g	
				Phenylalanine	6.0 g	6.0g^b^	5.70 g	
				Tyrosine	3.6 g		–	
Fat	1–2%	1.5%	1.92%	USFA	74.4%	–	–	Mitharwal et al. (2021)
				SFA	25.6%	–	317 mg	
				Palmitic acid	26.18%	–	290 mg	
				Oleic acid	–	–	585 mg	
				Stearic acid	50.43%	–	27.86 mg	
				Linoleic acid	0.12%	–	362 mg	
				Linolenic acid	20.26%	–	–	
Moisture	10–12%	12%	10.89%					
Micronutrients								
Water-soluble and fat-soluble vitamins				Thiamine (Vit. B1)	0.42 mg	0.42 mg	0.37 mg	Mitharwal et al. (2021)
				Riboflavin (Vit. B2)	0.19 mg	0.19 mg	0.17 mg	
				Niacin (Vit. B3)	1.10 mg	1.1 mg	1.34 mg	
				Retinol (Vit. A)	6 mg	–	–	
				α–tocopherol Vit. E	22 mg	–	–	
Minerals	1.7–4.13%	2.6%	2.04%	Calcium, Ca	344 mg	350 mg	364 mg	Thapliyal and Singh (2015)
				Potassium, K	408 mg	–	–	
				Phosphorous, P	130–250 mg	320 mg	–	
				Magnesium, Mg	137 mg	137 mg	–	
				Iron, Fe	3.3–14.8 mg	3.9 mg	4.62 mg	
				Sodium, Na	11–50 mg	–	–	
				Manganese, Mn	5.49 mg	5.49 mg	–	
				Zinc, Zn	2.30 mg	2.3 mg	–	
				Copper, C	0.47 mg	0.47 mg	0.67 mg	
Energy	1342 kJ	1405.82 kJ	1342 kJ					

Sources: *Respective References, **FAO. 1970, ***ICAR-IIMR (Rao et al. 2017), a is combined value for cysteine and methionine, and b is combined value for Phenylalanine and tyrosine

*USFA* unsaturated fatty acids, *SFA* saturated fatty acids

## Protein and amino acid profile

The protein content of Finger millet ranges from 5.0 to 8.0%. Prolamin is the predominant component of finer millet protein, accounting for 24.6–36.2% of total protein. It has a good amino acid profile with high amount of sulfur containing amino acids as well (Bhatt et al. 2003). The total essential amino acids in Finger millet are 44.7%, which is greater than the essential amino acids (33.9%) found in FAO reference protein (Mbithi-Mwikya et al. 2000). The higher protein content was related to higher prolamin content in the white varieties compared to brown varieties (Virupaksha et al. 1975). Finger millet is free of gluten protein, thus making it a good option for gluten-sensitive people.

## Carbohydrate and dietary fiber profile

Finger millet contains around 60.0% starch, 1.4–1.8% cellulose, and 0.04–0.6% lignins (Hassan et al. 2021) (Table 1). Amylopectin accounts for 80–85% of starch, while amylose makes up 15–20%. The starch granules are polygonal and rhombic in form. Non-starch polysaccharides (20–30% of total carbohydrate) contain 1.5% reducing sugar and 0.03% non-reducing sugar (Bhatt et al. 2003). Finger millet has 0.59–0.69 g of sugar per 100 g, with sucrose accounting for the majority (0.20–0.24/100 g) (Hassan et al. 2021). Finger millet contains a higher proportion of nutritional fibre than other cereals. Rathore et al. reported 18.6% dietary fibre and 4.3% crude fibres in Finger millet (Rathore et al. 2019). The overall dietary fibre content is 12%, made up of insoluble dietary fibre (11%) and soluble dietary fibre (2%) (Udeh et al. 2017). Finger millet's fibre content accumulates in the pericarp and endosperm walls. Dietary fibre advantages include colon cancer prevention, increased fecal bulk, increased fecal transit time, lowered blood lipids, and fermentability (He et al. 2022).

## Fat content and fatty acid profile

Finger millet has a low-fat content (1.3–1.8%), which contributes to its long shelf life. Unsaturated fatty acids account for the majority of total lipids (74.4%). Saturated fatty acids account for 25.6% of total lipid (Sridhar and Lakshminarayana 1994). Total lipid is divided into three categories: free (2.2%), bound (2.4%), and structural lipids (0.6%). Oleic acid, palmitic acid, and linoleic acid are the most abundant fatty acids, accounting for 50.43%, 26.18%, and 20.26%, respectively. However, stearic acid and linolenic acids are present in lower concentrations, at 0.12% and 2.60%, respectively (Mitharwal et al. 2021) (Table 1).

## Vitamins and minerals profile

Finger millet is high in both water-soluble and fat-soluble vitamins, which are necessary for proper cell division and brain function (Devi et al. 2014). It contains 6 mg of retinol (vitamin A), 0.42 mg of thiamine (B1), 0.19 mg of riboflavin (B2), 1.10 mg of niacin (B3), and 22 mg of tocopherol (E) per 100 g. Finger millet contains 35%, 12%, and 8% of the recommended daily allowance (RDA) for healthy individuals (Pragya and Rita 2012; Mitharwal et al. 2021).

Finger millet is rich in minerals, mainly present in the pericarp, aleurone layer, and germ. According to Thapliyal and Singh, Finger millet has a total ash content of 1.7–4.13%. It contains calcium (344 mg/100 g of Finger millet), potassium (408 mg/100 g of Finger millet), phosphorus (130–250 mg/100 g of Finger millet), magnesium (137 mg/100 g of Finger millet), and iron (3.3–14.8 mg/100 g of Finger millet) (Thapliyal and Singh 2015). The iron content of the 16 distinct Finger millet types ranged from 3.61 to 5.42 mg per 100 g (Singh and Srivastava 2006).

Table 1 summarizes the macronutrient (carbohydrates, proteins, fats, dietary fiber) and micronutrient (vitamins, minerals) content in Finger millet, with values from different research studies, and organizations including FAO and ICAR-IIMR.

## Nutritional comparison of Finger millet with other cereals

The nutritional comparison of Finger millet with other major and minor millets and cereals is listed in Table 2A–C. Finger millet is rich in dietary fiber (~ 11.5%) which is higher than brown rice, polished rice, and other millets, such as kodo millet, barnyard millet, little millet, and foxtail millet. Finger millet has a protein content comparable to rice (~ 7.9%), but lower than wheat and sorghum (Arya and Bisht 2022). When compared to other grains, Finger millet provides more than 40% of the essential amino acids (methionine, leucine, isoleucine, tryptophan, phenylalanine, and threonine) (Ramashia et al. 2019). Finger millet has a greater methionine concentration (194 mg/100gm) than other cereals and millet (Maharajan et al. 2021). These necessary amino acids help to reduce the risk of obesity, cancer, and excessive cholesterol. Unlike other grains, lysine is not a limiting amino acid in Finger millet. It also contains a high level of tryptophan and threonine, which are lacking in rice, wheat, and sorghum. Apart from having higher nutritional value than wheat, rye, barley, and oats; Finger millet grains are gluten-free, making them easy to digest and non-allergenic to celiac disease patients (Saxena et al. 2018). Finger millet has a fat level of 1–2%, which is lower than other cereal grains (3.5–5.2% fat) (Shahidi and Chandrasekara 2013). Finger millet has better keeping quality than minor cereals, such as barnyard millet, foxtail millet, and pearl millet, because it contains less fat (Rathore et al. 2019). When compared to other cereals, such as wheat, maize, or brown rice, Finger millet has the highest calcium (344 mg/100 g) and potassium content (408 mg/100 g). It contains ten times more calcium than rice and wheat, and three times more than milk (Shibairo et al. 2016). Furthermore, the crude fibre and mineral content (15–20%, 2.5–3%, respectively) are higher than rice (0.2% and 0.6%, respectively) and wheat (1.2% and 1.5%). Finger millet contains more calcium (398 mg/100 g), potassium (430–490 mg/100 g), phosphorus (130–283 mg/100 g), and iron (3–20%) than other millets (Manjula et al. 2015; Jayawardana et al. 2019).

**Table 2:** Nutritional comparison of Finger millet with other cereals

A. Proximate composition analysis (g/100 g or %)								
Components	Finger millet	Little millet	Kodo millet	Sorghum	Pearl millet	Wheat	Raw milled rice	Maize
Protein	7.16	8.92	8.92	9.97	10.96	10.59	7.94	8.80
Total Fat	1.92	2.55	2.55	1.73	5.43	1.47	0.52	3.77
Dietary fiber								
Total	11.18	6.39	6.39	10.22	11.49	11.23	2.81	12.24
Insoluble	9.51	5.45	4.29	8.49	9.14	9.63	1.99	11.29
Soluble	1.67	2.27	2.11	1.73	2.34	1.60	0.82	0.94
Carbohydrates	66.82	65.55	66.19	67.68	61.78	64.72	78.24	64.77
Ash	2.04	1.72	1.72	1.39	1.37	1.42	0.56	1.17
Moisture	10.89	14.23	14.23	9.01	8.97	10.58	9.93	9.26
Energy (KJ)	1342	1449	1386	1398	1456	1347	1491	1396

B. Water-soluble and fat-soluble vitamin and mineral profile								
Components	Finger millet	Little millet	Kodo millet	Sorghum	Pearl millet	Wheat	Raw milled rice	Maize
Water-soluble vitamin								
Thiamine-B1 (mg)	0.37	0.26	0.29	0.35	0.25	0.46	0.05	0.33
Riboflavin-B2 (mg)	0.17	0.05	0.20	0.14	0.20	0.15	0.05	0.09
Niacin-B3 (mg)	1.34	1.29	1.49	2.10	0.86	2.66	1069	2.69
Pantothenic acid-B5 (mg)	0.29	0.60	0.63	0.27	0.50	1.08	0.57	034
Total B6 (mg)	0.05	0.04	0.07	0.28	0.27	0.26	0.12	0.34
Biotin-B7 (μg)	0.88	6.03	1.49	0.70	0.64	1.03	0.60	049
Total folate (μg)	34.66	36.20	39.49	39.42	36.11	30.09	9.32	25.81
Fat-soluble vitamins								
α-tocopherol (mg)	0.16	0.55	0.07	0.06	0.24	0.77	0.06	2.50
Phylloquinones-K (μg)	3.00	4.47	0.06	43.82	2.85	1.7	1.50	2.50
Minerals (mg/100 g)								
Calcium	364	16.06	15.27	27.60	27.35	39.36	7.49	2.82
Iron	4.62	1.26	2.34	3.95	6.42	3.97	0.002	2.49
Phosphorus	341		188	350	296	1170		990

C. Carbohydrate and fatty acid profile								
Components	Finger millet	Little millet	Kodo millet	Sorghum	Pearl millet	Wheat	Raw milled rice	Maize
Carbohydrates								
Total available carbohydrate (g)	62.47	56.43	66.25	60.96	56.02	58.60	76.39	61.01
Total starch (g)	62.13	56.07	64.96	59.70	55.21	56.82	75.70	59.35
Total free sugars (g)	0.34	0.37	1.29	1.27	0.81	1.60	0.69	1.66
Fatty acids								
Palmitic (mg)	290	487	211	149	729	176	143	363
Stearic (mg)	27.86	102	28.40	14.22	128	14.83	14.50	42.45
Oleic (mg)	585	868	291	3.14	1040	141	109	700
Linoleic (mg)	362	1230	576	508	1844	616	234	1565

*Source: (Abioye et al. 2022), Indian Food Composition Tables, NIN-2017 and Nutritive value of Indian foods, NIN-2007

## Phytochemical/bioactive compounds profile

Phytochemicals are a diverse group of biologically and physiologically active compounds with positive health effects. The research suggests that vegetables, fruits, legumes, nuts, and whole grains provide health benefits due to their high phytochemical content (Panche et al. 2016). Finger millet is important among minor grains due to its high concentration of beneficial food components, particularly polyphenols and dietary fibre (Table 3) (Chandra et al. 2016; Balasubramaniam et al. 2019). Finger millet features dark brown seeds that are higher in polyphenols than wheat, rice, maize, and barley.

**Table 3:** Phytochemical profile of Finger millet

Phytochemicals	Content	References
Total polyphenols	10.2 mg/100 g	Hithamani and Srinivasan (2014)
Phenolic acid	0.39–1.05%	Lansakara et al. (2016)
Tannins	340–500 mg/100 g	Nakarani et al. (2021)
Flavonoid	62.23–74.05 mg/100 g	
Trypsin inhibitor	207.35–234.23 mg/100 g	
HCN	2.45–2.80 mg/100 g	
Phytate	210.75–302.75 mg/100 g	
Oxalate	19.80–26.23 mg/100 g	

Polyphenols are abundant in the bran (seed coat tissue), contributing for up to 60% of total polyphenols (Kumar et al. 2016). The phenolic component in the seed coat is 0.8%, while it accounts for 6.2% of the flour. Polyphenols are mostly composed of phenolic acid and tannins, with flavonoids accounting for a minor fraction. Phenolic acids and tannins help the body defend against oxidative stress (Onyekere et al. 2018). Finger millet includes a variety of phenolic components, including major bound phenolic (ferulic acid, p-coumaric acid), major free phenolic (proto-catechuic acid), and tannins. The varietal differences in the polyphenol content among the 32 varieties of Finger millets revealed the polyphenol content of 1.2–2.3% in the brown varieties and 0.3–0.55% in white varieties (Ramachandra et al. 1977). Polyphenol content ranged between 0.54 and 3.4% in African types and 0.08 to 0.96% in Indian variants. Another study found that the total phenolic content of two varieties of Finger millets ranged between 0.39 and 1.05 mg of catechin equivalents/g (Priyanwada et al. 2020). The total phenol concentration of the ten distinct Finger millet genotypes ranged from 99.75 to 112.25 mg/100 g, while the phytic acid level ranged from 210.75 to 302.75 mg/100 g. Tannin content ranged from 340 to 500 mg/100 g, while flavonoid levels ranged between 62.23 and 74.05 mg/100 g. Parida et al. discovered that white Finger millet variants had a lower tannin level (0.05%) than brown types. The tannin concentration in two African types, IE927 and IE929, ranged from 3.42 to 3.47% (Parida et al. 1989). Chethan and Malleshi (2007) found 1.3–2.3% total polyphenols in five brown variants and 0.3–0.5% total polyphenols in two white kinds. The native Finger millet had a total polyphenol concentration of 10.2 mg/g, according to Hithamani and Srinivasan (Hithamani and Srinivasan 2014). Lansakara et al. (2016) found total phenolic acid in the range of 0.39–1.05% (mg Catechin equivalent/g) in two Finger millet types, Ravana and Osada. The use of different extraction procedures resulted in variations in polyphenol contents (Banerjee et al. 2012). High quantities of tannin function as a barrier to fungal infection, protecting grains from fungal infections. Major flavonoids include quercetin, catechin, epicatechin, gallocatechin, epigallocatechin, condensed tannins, and proanthocyanidins (Udeh et al. 2017). Catechin and epicatechin dominate the free fraction of Finger millet, whereas ferulic acid and trans-p-coumaric acid dominate the bound fraction (Xiang et al. 2018).

The antioxidant properties of four white, red, brown, and reddish types were assessed. The brown type had the highest total antioxidant content in terms of phenolic acid, flavonoids, and catechin tannin, whereas the white variety had the lowest. The abundance of polyphenols and carotenoids in coloured cultivars confers the highest total antioxidant properties (Thapliyal and Singh 2015). The contents of phenolic acid varied from 292.29 to 302.42 mg ferric acid eq/100 g, total flavonoids ranged from 90.24 to 202.94 mg catechin acid eq/100 g, and total catechins ranged from 31.76 to 83.59 mg (Xiang et al. 2018). Two Sri Lankan cultivars, Ravana and Oshada, were tested for phytochemical properties. The Oshada variety contained more flavonoids (1.05 mg/GAE/g) and total phenolics (8.08 mg/GAE/g) than the Ravana variety (Lansakara et al. 2016). The phenolic content of two Sri Lankan cultivars, Bala and Wadimal, was estimated to be 160.20–181.39 mg GAE/100 g (Jayawardana et al. 2019). Ascorbic acid promotes iron absorption and immunity. Chen et al. found that some Indian Finger millet cultivars, including VL 324 (54.49 µg/g) and VL204 (64.92 µg/g), contain ascorbic acid (Chen et al. 2003).

Some phytochemicals, such as phytates, tannins, trypsin inhibitors, and oxalates, have been identified as anti-nutritional factors due to their metal chelating and enzyme inhibitory properties (Devi et al. 2014). Other antinutrients include trypsin inhibitor (207.35–234.23 mg/100 g), HCN (2.45–2.80 mg/100 g), phytate (240–300 mg/100 g), and oxalate (19.80–26.23 mg/100 g) (Nakarani et al. 2021). The content of phytochemicals varies according to the processing and extraction processes used (Table 4). Chethan and Malleshi discovered that acidic methanol extraction effectively retrieved polyphenols from Finger millet, with stability observed at acidic-to-neutral pH levels but declining at alkaline pH. High-performance liquid chromatography (HPLC) analysis identified benzoic acid (gallic acid, p-hydroxy benzoic acid, proto-catechuic acid) and cinnamic acid (syringic acid, ferulic acid, p-coumaric acid, trans-cinnamic acid), with gallic acid and ferulic acid levels decreasing as pH rose from 3 to 10 (Chethan and Malleshi 2007). Balasubramaniam et al. compared ultrasonication (UA), enzyme pretreatment in ultrasonication (EUA), and heat reflux (HR) for extracting polyphenols from Finger millet seed coats. EUA with xylanase (XUA) significantly enhanced phenolic content by about twofold, flavonoids by 1.3, and tannins by 1.2, marking the first use of green technology for such extraction. UA accelerates molecular collision via ultrasonic power, while EUA disrupts plant cell walls through enzymatic action, improving phenolic recovery without thermal degradation (Balasubramaniam et al. 2019). Xiang et al. extracted phenolic compounds from Finger millet using 80% methanol, finding dark-colored cultivars richer in phenolic acid than light-colored ones. Brown and reddish varieties exhibited higher total phenolic content (~ 302 mg ferulic acid equivalent/100 g, DW; ~ 292 mg ferulic acid equivalent/100 g, DW) compared to the white variety (~ 172 mg ferulic acid equivalent/100 g, DW) (Xiang et al. 2018). Polyphenol intake and bioavailability significantly affect consumer health, as higher bioavailability enhances absorption. Food matrix breakdown by digestive enzymes and microbiota in the intestines determines polyphenol bioaccessibility, influenced by both food composition and processing methods in Finger millet. Hithamani et al. explored how domestic processing affects polyphenol bioaccessibility in Finger millet. They observed that sprouting and roasting significantly enhanced phenolic bioaccessibility, increasing it by 67%. Conversely, pressure cooking, open-pan boiling, and microwave-heating led to a significant reduction of 30–35% in bioaccessibility (Hithamani and Srinivasan 2014).

**Table 4:** Effect of different extraction or processing methods on phytochemical contents of Finger millet

Extraction or processing methods	Effect on phytochemical content	References
Acidic methanol extraction (1% HCl-method) at different pH and temperature	Polyphenol content was independent of temperature Total phenolic content decreased from 6.4% to 2.5% at pH 3 and pH 10 respectively	Chethan and Malleshi (2007)
Ultrasonication (UA) and enzyme pretreatment in the utrasonication (EUA	Twofold increase in phenolic content 1.3-fold increase in flavonoids 1.2-fold increase in tannins by EUA with xylanase (XUA)	Balasubramaniam et al. (2019)
Use of 80% methanol	The total phenolic content of brown and reddish varieties was ~ 302 mg FAE/100 g DW and ~ 292 mg FAE /100 g DW which is higher than the total phenolic content of white variety i.e. ~ 172 mg FAE/100 g	Xiang et al. (2018)
Pressure cooking, open-pan boiling, and microwave-heating and sprouting	Bioaccessibility of polyphenols reduced by 30–35% by pressure cooking, open-pan boiling, and microwave-heating while sprouting increased the bioaccessibility of polyphenols by 67%	Hithamani and Srinivasan (2014)

*FAE* ferulic acid equivalent, *DW* dry weight

## Biological activities of Finger millet

### Antidiabetic

Diabetes mellitus is a chronic metabolic disorder with a rapidly increasing prevalence, not only in India but also in many other nations. Diabetes is characterized by elevated levels of blood glucose, known as hyperglycemia (International Diabetes Federation 2021). While it is important to control fasting blood glucose, addressing the rise in blood sugar after meals is especially significant, because elevated postprandial glucose levels can contribute to various complications associated with diabetes (Hershon et al. 2019). Chemical synthetic inhibitors of α-glucosidase and pancreatic amylase are commonly used in diabetes management managing postprandial hyperglycemia. These inhibitors slow down the digestion of carbohydrates, leading to a more gradual release of glucose into the bloodstream. The phenolic extracts obtained from the seed coats of Finger millet are known to exhibit inhibitory properties against these enzymes. Consuming meals high in fibre and complex carbohydrates helps prevent subsequent blood glucose spikes, which is essential for managing diabetes and lowers chronic vascular issues. A potential link between the intake of dietary calcium and magnesium and a reduced risk of type-2 diabetes has been indicated by two independent studies (Pittas et al. 2006; Van Dam et al. 2006). Finger millet’s richness in these minerals may be one factor among several contributing to its potential role in reducing the risk of type-2 diabetes. The advantages of cereal grains were related to its dietary fibre and polyphenols contents which were recognized to lower the incidence of diabetes mellitus (Devi et al. 2014) and gastrointestinal tract diseases.

A study found that consuming multigrain flour with a 30% proportion of Finger millet significantly reduced plasma glucose levels. This effect was attributed to the slowed digestion of carbohydrates, likely facilitated by dietary fiber. The inhibition of α-amylase and α-glucosidase enzymes by phenolics is a key mechanism that contributes to their impact on postprandial blood glucose concentrations (Chethan et al. 2008; Pradhan et al. 2010). Their ability to inhibit the activity of digestive enzymes such as α-amylase, lipases, trypsin, pepsin, α-glucosidase, among others, is one of the mechanisms through which they exert their effects on postprandial hyperglycemia (Rohn et al. 2002). Antinutritional factors, such as tannins, phenolics, and phytates when present in whole Finger millet fractions, may play a role in reducing the glycemic response (Kumari and Sumathi 2002).

Hegde et al. investigated the effects of Finger millet whole-grain flour on alloxan-induced type II diabetes rats over 28 days. Rats fed with this flour experienced decreased cholesterol and blood glucose levels (13% and 36%, respectively) alongside restored antioxidant levels and reduced lipid peroxide, thus suggesting potential benefits in managing oxidative stress and blood glucose in diabetes (Hegde et al. 2002). Studies suggest that certain compounds in Finger millet may influence insulin sensitivity, which is crucial in type 2 diabetes management (Lakshmi Kumari and Sumathi 2002). Foods containing Finger millet have shown to have a lower glycemic index and result in a reduced glycemic response upon consumption compared to certain other grains and refined carbohydrates (Shobana et al. 2009; Shukla and Srivastava 2014). Finger millet (GI = 61.1 ± 10.3) is more effective than milled rice (71.7 ± 14.4) and refined wheat (74.2 ± 14.9) in terms of reducing the glycemic index (Anitha et al. 2021). Diabetes can be associated with various complications and delayed wound healing and increased risk of cataracts. Finger millet diet had a positive impact on the speed of healing for skin wounds and delayed the onset of cataracts in the rat models (Rajasekaran et al. 2004; Shobana et al. 2010). Epidemiological reports have shown a lower incidence of diabetes and its complications in millet eating communities (Saleh et al. 2013). A study investigated the anti-diabetic and antioxidant activities of four millet types in South Korea. Finger Italian millet exhibited the highest anti-diabetic activity in vitro, with lower IC50 values for α-glucosidase and α-amylase compared to the standard drug acarbose (Ofosu et al. 2020).

### Antioxidant and anti-ageing

Dietary plant polyphenols have indeed gained significant attention from health professionals, nutrition scientists, and consumers due to their multifaceted health benefits (Kaur and Kapoor 2001; Scalbert et al. 2005; Tsao 2010). Finger millet is known for its rich content of various phenolic compounds (mostly derivatives of benzoic acid), particularly in the seed coat. Proto-catechuic acid (45.0 mg/100 g) has been reported as the major free phenolic acid in Finger millet grains (Rao and Muralikrishna 2007). A diet consisting of 55% Finger millet led to increased activities of antioxidant enzymes specifically, catalase, glutathione peroxidase, and glutathione reductase in rats implying a protective role of Finger millet in terms of antioxidant defense (Hegde et al. 2005). Finger millet predominantly contains benzoic acid derivatives and smaller fractions of cinnamic acid derivatives and flavonoids (Chethan and Malleshi 2007). Ferulic and p-coumaric acids constitute a significant portion of the bound phenolic fraction in Finger millet grains (Devi et al. 2014).

Finger millet has the potential to inhibit collagen cross-linking, a process that contributes to the stiffness and reduced elasticity of tissues, including tendons, skin, and blood vessels (Hegde et al. 2002). The processing of Finger millet, including thermal or hydrothermal treatments, germination, decortication, or fermentation, led to reduction in polyphenol levels often leading to a diminished radical quenching ability compared to the unprocessed grain (Towo et al. 2003; Rao and Muralikrishna 2007; Shobana and Malleshi 2007). Recent findings reflected that Ravi, Rawana, and Oshadha Sri Lankan Finger millet varieties are good sources of antioxidants (Jayawardana et al. 2021).

### Anti-carcinogenic

The inclusion of certain foods with anti-carcinogenic properties in the diet may influence the frequency or rate of spontaneous or induced tumors, potentially reducing the risk of cancer. A protease inhibitor isolated and purified from ragi seeds is a well-characterised protease inhibitor, popularly known as “Ragi Bifunctional Inhibitor (RBI)”. RBI, a 14 kDa bifunctional inhibitor, belongs to the cereal trypsin/α-amylase inhibitor family and functions as a bifunctional inhibitor, which can inhibit both α-amylase and trypsin (Maskos et al. 1996). RBI is a single polypeptide chain consisting of 122 amino acids, and it incorporates five intramolecular disulfide bonds, contributing to its overall stability and functionality (Campos and Richardson 1983). Sen and Dutta investigated the anti-carcinogenic activity of RBI from Finger millet seeds against K562 human chronic myeloid leukemia cells. RBI demonstrated suppression of proliferation and induction of apoptosis specifically in K562 cells, showing selectivity towards cancer cells over normal human cells in vitro (Sen and Dutta 2012).

Phytochemicals and antioxidants are two important nutraceutical components found in various plant-based foods, and they are known for their extensive anti-carcinogenic properties (Shin et al. 2018; Di Gioia et al. 2020). The diverse array of compounds found in Finger millet, including phytochemicals and antioxidants, suggests that it may have the potential to suppress excessive cellular oxidation. This, in turn, could provide protection from different types of cancers that are prevalent in the human population. Ferulic acid has been reported to exhibit a blocking effect on induced carcinogenesis in the tongue and colon of rats (Mori et al. 1999; Kawabata et al. 2000) and as a natural bioactive chemotherapeutic agent against breast cancer cells (Choi and Park 2015). This study suggests that ferulic acid, being a major constituent of bound phenolic acids in Finger millet, may have potential as a natural bioactive chemotherapeutic agent against cancer. It has been reported that populations consuming sorghum and millet had lower incidences of oesophageal cancer compared to those consuming wheat or maize (Chen et al. 1993). There are many evidences that have established the inverse relation between whole cereal-based dietary fiber intake and breast cancer risks (Khan et al. 2012; Mourouti et al. 2016; Dreher 2018) implicating that whole grains might hold nutraceutical characteristics against breast cancer. The extracted compounds from Finger millet variety KMR 301 using 70% ethanol and 10% alkali seeds, specifically free phenolic acids, demonstrated anti-cancer effects by inducing cell death in breast and colorectal cancer cells. The mechanisms involved cell cycle arrest, DNA fragmentation, and the accumulation of cells in the Sub-G1 phase (Saleh et al. 2013; Kuruburu et al. 2022). Dietary fiber from Finger millet, particularly its phenolic compounds, holds promise in potentially suppressing the growth of various neoplasms, including breast and colorectal cancers (Mahadevaswamy et al. 2022).

### Cardio-protective

Cardiovascular diseases are a major global health concern, contributing significantly to morbidity and mortality across diverse populations (Zhao et al. 2021). Abnormal blood pressure, elevated cholesterol, hypertension or depression, obesity, and diabetes are important risk factors that contribute to the aggravation of cardiovascular diseases. Finger millet has positive effect on hyperlipidemia in rats by reducing triglycerides and total cholesterol in their serum (Lee et al. 2010). Diet containing Finger millet constituents may lead to lower lipid peroxidation and helps reduce arteriosclerosis implying a positive impact on cardiovascular health. The diet with Finger millet as one of the constituents had an impact on control lipid metabolism and antioxidant metabolism (Vasant et al. 2014). Phenolic extracts from various millets have been studied for their impact on the oxidative modification of low-density lipoprotein (LDL) cholesterol in vitro system involving copper to induce oxidative modification of LDL cholesterol (Chandrasekara and Shahidi 2012a) Soluble dietary fiber component of the grain plays a role in reducing the reabsorption of bile acids, which are synthesized from cholesterol, and subsequently contributes to a decrease in LDL cholesterol (Chandrasekara and Shahidi 2012a; Shahidi and Chandrasekara 2013). Fermentation of Finger millet presents a cost-effective means to produce statin and sterol metabolites, useful in managing hypercholesterolemia. These metabolites, particularly statins, act as inhibitors of HMG-CoA reductase, a key enzyme in cholesterol biosynthesis, highlighting Finger millet's potential in cholesterol management (Venkateswaran and Vijayalakshmi 2010).

### Antimicrobial

Traditional methods of food preservation often relied on synthetic chemicals as antimicrobial agents. However, there has been a growing interest and emphasis on exploring natural products with antimicrobial properties for food preservation. With phenolic acids from different fractions of Finger millet, specifically from whole flour, seed coat was isolated and evaluated for their antimicrobial properties. Interestingly, the seed coat extract demonstrated higher antimicrobial activity against *Bacillus cereus* and *Aspergillus flavus* (Varsha et al. 2009)*.* Phenolic content and flavonoids in Finger millet inhibit microbial membranes and enzymes, targeting bacteria, such as *Escherichia coli, Bacillus cereus, Listeria monocytogenes, Staphylococcus aureus, Streptococcus pyogenes, Serratia marcescens, Proteus mirabilis, Pseudomonas aeruginosa, Klebsiella pneumoniae*, and *Yersinia enterocolitica* (Banerjee et al. 2012)*.* Finger millet polyphenols demonstrate antimicrobial activity against a range of pathogens including *E. coli, S. aureus, P. mirabilis, P. aeruginosa, S. marcescens, K. pneumoniae, Shigella dysenteriae, Enterococcus spp.,* and *Salmonella spp.* (Singh et al. 2015)*.* Antibacterial and β-lactamase enzyme inhibitory activities of ethanolic and methanolic extracts of Sri Lankan Finger millet varieties against antibiotic-sensitive S*. aureus* (ATCC^®^ 6538™) and Bacillus subtilis (ATCC^®^ 23857™) strains (Jayawardana et al. 2020).

### Osteoporosis

Osteoporosis is indeed a progressive bone disease characterized by various factors that collectively lead to weakened and brittle bones, making them more susceptible to fractures The World Health Organization (WHO) has recognized osteoporosis as a significant global health concern. Osteoporosis is influenced by various factors, and both dietary calcium and vitamin D play crucial roles in bone health. Finger millet emerges as a promising alternative to conventional calcium supplements, with its seeds containing a notable calcium content of 364 ± 58 mg/100 g. This surpasses the calcium content found in milk (476 mg/100 g) and offers a potentially affordable and side-effect-free source of this essential mineral (Singh and Raghuvanshi 2012; Bhavya Bhanu et al. 2017; Palacios et al. 2021). Consuming 100 g of Finger millet can contribute approximately half of the RDA for calcium, assuming an average calcium content of 324.5 mg/100 g. Also, high cost of milk impacts its intake, especially among young children and pregnant women from underprivileged socioeconomic segments in India (Kumar et al. 2014). Moreover, there is a high prevalence of lactose intolerance, a common condition characterized by the inability to fully digest lactose, a sugar found in milk and dairy products (Hodges et al. 2019). Finger millet emerges as a valuable alternative staple with high-calcium content, thus offering a promising solution to nutritional gaps particularly for lactose-intolerant populations. Its underexplored potential in diverse diets, especially in developing countries, suggests significant benefits for addressing calcium-related health concerns and promoting sustainable nutrition through public health initiatives and dietary recommendations. Table 5 provides the summarised biological activities of finger millet.

**Table 5:** Summarised biological activities of Finger millet

Properties	Functional role	References
Antidiabetic properties	(i) Phenolic compounds in whole-grain flour—controlling blood glucose levels in alloxan-induced type II diabetes rats	Hegde et al. (2005)
	(ii) Impact on postprandial blood glucose concentrations, reduce the risk of diabetes induced cataract diseases	Chethan et al. (2008)
	(iii) Phenolics extract from the seed coat inhibits α-glucosidase and pancreatic amylase reduce postprandial hyperglycemia by partially inhibiting the enzymatic hydrolysis of complex carbohydrates	Shobana et al. (2009)
	(iv) Multigrain flour containing 30% Finger millet proportion: lowered plasma glucose levels due to delayed carbohydrate digestibility mediated by dietary fiber	Pradhan et al. (2010)
	(v) Positive impact on the speed of healing for skin wounds and delayed the onset of cataracts in the rat models	Rao and Muralikrishna (2002), Rajasekaran et al. (2004), Shobana et al.( 2010) and Shukla and Srivastava (2014)
	(vi) Finger millet-based foods- exhibit a lower glycemic index and contribute to a lower glycemic response	
Antioxidant properties	(i) Seed coat—possess antioxidant activity	Hegde et al. (2005) and Chandrasekara and Shahidi (2010)
	(ii) Seed coat/acidic methanol—derivatives of benzoic acid (gallic acid, proto-catechuic acid, and *p*-hydroxy benzoic acid) and cinnamic acid (*p*-coumaric acid, syringic acid, ferulic acid, and *trans*-cinnamic acid)	Chethan et al. (2008)
	(iii) Whole flour methanol extract—antioxidant activity, linoleic acid assay, DPPH radical, hydroxyl quenching action	Varsha et al. (2009)
	(iv) Ferulic and p-coumaric acid—bound phenolic fraction in Finger millet accounting for 64– 96 and 50–99% of total ferulic and p-coumaric acid content of Finger millet grains	Devi et al. (2014)
Anti-carcinogenic properties	(i) Phenolic components—tannins, and phytate—help in reducing cancer initiation and progression	Chandrasekara and Shahidi (2011a, b)
	(ii) Phenolics and phenolic acid derivatives, flavonoids, and aminoacids in the free (FM-FP) and bound (FM-BP) phenolic compounds- modulate the proliferative potential of breast and colorectal cancer cells	Mahadevaswamy et al. (2022)
	(iii) Finger millet variety KMR 301—demonstrated anti-cancer effects by inducing cell death in breast and colorectal cancer cells	Kuruburu et al. (2022)
Cardio-protective properties	(i) Fermentation of Finger millet: produce metabolites like statin and sterol—employed in therapies designed to address hypercholesterolemia	Venkateswaran and Vijayalakshmi (2010)
	(ii) Phenolics: oxidative modification of LDL cholesterol in a vitro system involving copper to induce oxidative modification of LDL cholesterol	Chandrasekara and Shahidi (2012a, b)
	(iii) Soluble dietary fibre: reduces the reabsorption of bile acids	Chandrasekara and Shahidi (2012b)
	(iv) Control lipid metabolism and antioxidant metabolism	Vasant et al. (2014)
Antimicrobial properties	(i) Germinated and ungerminated millet phenol extract—against *Bacillus cereus, Staphylococcus aureus, Yersinia enterocolitica, Escherichia coli, Listeria monocytogenes, Streptococcus pyogenes, Pseudomonas aeruginosa, Serrtia marcescens, Klebsiella pneumonia*	Chethan and Malleshi (2007)
	(ii) Seed coat phenolic extract—active against *Bacillus cereus*, *Aspergillus niger*	Varsha et al. (2009)
	(iii) Seed coat rich fraction: active against *E. coli, B. cereus, Listeria monocytogenes, Staphylococcus aureus, Streptococcus pyogenes, Serratia marcescens, Proteus mirabilis, Pseudomonas aeruginosa, Klebsiella pneumoniae,* and* Yersinia enterocolitica*	Banerjee et al. (2012)
	(iv) Seed extract in ethyl acetate and hexane: active against *E. coli, Staphylococcus aureus, Proteus mirabilis, Pseudomonas aeruginosa, Serratia marcescens, Klebsiella pneumoniae, Shigella dysenteriae, Enterococcus sp., and Salmonella sp*	Singh et al. (2015)
	(v) Ethanolic and methanolic extracts of Sri Lankan Finger millet varieties: against antibiotic-sensitive S. aureus (ATCC® 6538™) and B. subtilis (ATCC® 23,857™) strains	Jayawardana et al. (2020)
Osteoporosis	(i) Finger millet seeds: Calcium content 364 ± 58 mg/100 g	Bhavya Bhanu et al. (2017)

## Industrial applications of Finger millet

### Functional ingredient in food industry

**Fig. 2:**
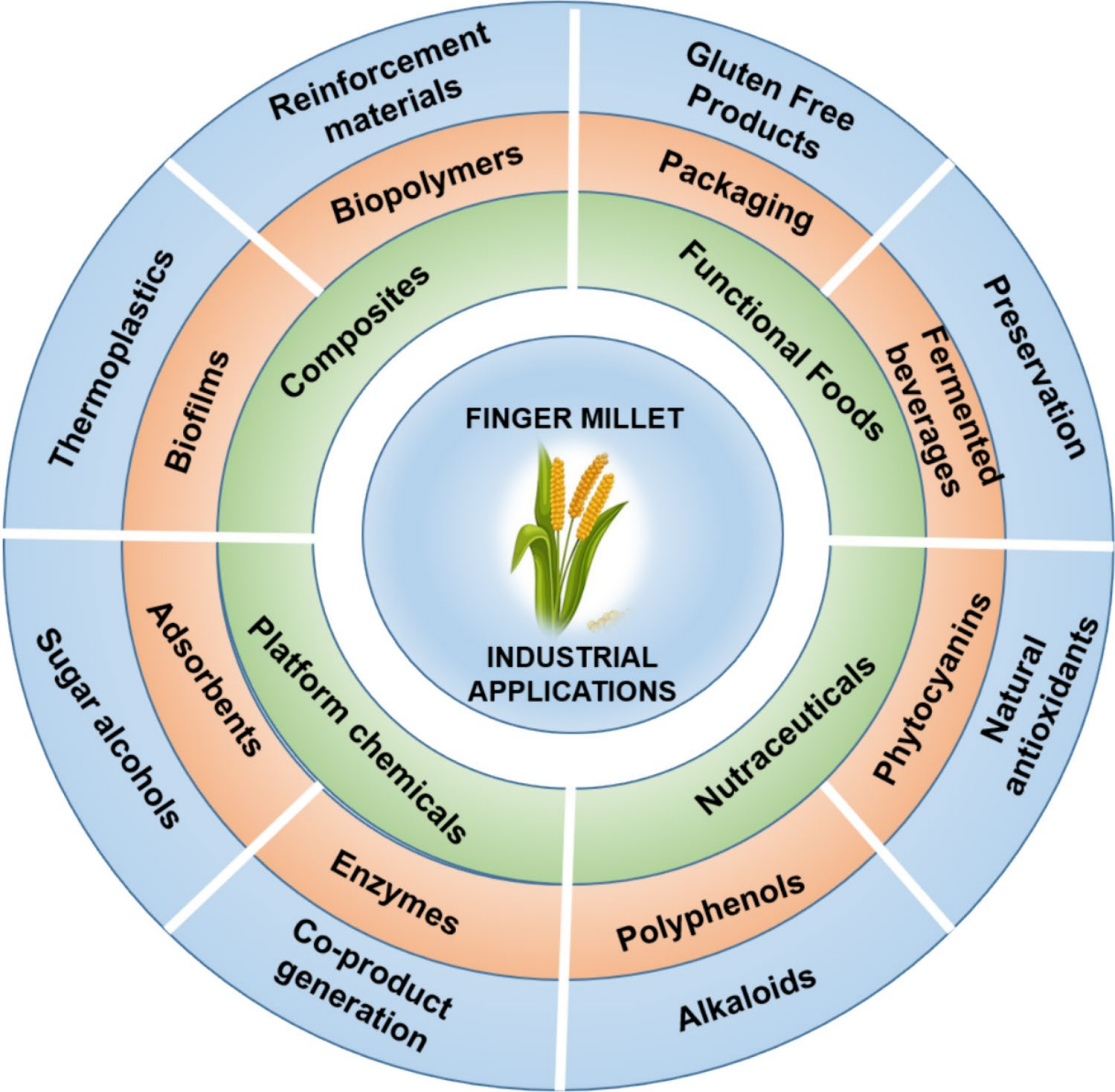
Industrial applications of Finger millet

Due to the avoidance of gluten containing products, the consumption of gluten-free cereals (GFCs) has increased substantially. The gluten-sensitive population has shifted to the diets that contain cereals that are without gluten or contain gluten less than 20 parts per million (Selladurai et al. 2023). It has been estimated that the global consumption of gluten-free products is expanding at a rate of 7.6% between the period 2020 and 2027 (Fajardo et al. 2020). The Finger millet grains are subjected to different processing conditions prior to consumption to remove inedible components and enhance their nutritive quality. The primary millet processing methods include soaking, germination, blanching, dry heat treatment/roasting, and milling. These methods improve the quality of the grains, thereby providing suitable product for human consumption. The secondary millet processing methods, which include fermentation, popping, malting, etc., are employed to produce various value-added products from Finger millet (Mitharwal et al. 2021; Selladurai et al. 2023). In a broader sense, the food products developed from Finger millet grains can be classified into conventional (pancakes, flatbread/roti; porridges/dalia; fermented items: masa, injera, dosa, idli, etc.) and non-conventional (baked items: cakes, muffins, biscuits, waffles, nan khatai, rusk; popped/puffed snacks and flakes; beverages: Koozh, komoni, kiambule; extruded items: spaghetti, macaroni, etc.) (Singh et al. 2013; Devi et al. 2014; Kubo 2016; Kumar et al. 2020). Various researchers have explored the avenue of value-added food production from Finger millet for improved nutritional composition and nutraceutical properties that have been highlighted in Fig. 2.

Further, the lignocellulosic material of Finger millet straw and husk shows a great potential in the development of polymer composite materials for various applications, including food packaging. These agricultural residues are used as reinforcement materials with polystyrene, polypropylene, polyvinyl acetate, polylactic acid, etc. to develop low-cost thermoplastic polymers with enhanced elastic modulus (Mamun and Bledzki 2013; Haque et al. 2021). Extracted husk fibers from Finger millet and developed thermoplastic composite using injection molding process. The composite presented considerable mechanical properties (maximum tensile strength: 30.28 MPa, tensile modulus: 860.06 MPa, flexural strength: 61.43 MPa, and flexural modulus: 2412.93 MPa) at 5% Finger millet husk loading. In another study, high-toughness epoxy biocomposite was synthesized using biosilica particles extracted from Finger husk reinforced with coconut rachilla fibre for food packaging purposes (Sivamurugan et al. 2024). The composite displayed excellent impact strength, tensile strength, flexural strength, and hardness mechanical.

To extend the shelf life of food products and prevent microbial/moisture-related damage, edible and sustainable films are used for food packaging (Sharma et al. 2023). The underutilized Indian Finger millet starch was utilized by Gautam et al. to develop flexible and thin films supplemented with sorbitol (used as plasticizer) for improved mechanical and thermal properties. The results in this study demonstrated that native Finger millet starch can be useful in the development of edible films and coatings (Gautam et al. 2022). The effect of plasticization of glycerol and sorbitol was studied for upgrading the properties of Finger millet starch-based film. It was reported that the glycerol-based film matrix exhibited better thermal stability, compactness, and homogeneity in comparison to sorbitol-based film (Sharma et al. 2023). A study prepared chitosan/gelatin-based films by incorporating Finger millet bran extract (FMBE) at different concentrations. At a concentration of 0.5%, the addition of FMBE significantly improved biological and physico-chemical characteristics of the chitosan/gelatin films prepared for food packaging and preservation. Further, salad dressings and high-fat food items (such as mayonnaise, etc.) produced from polyphenols and other natural antioxidants play a prominent role in food preservation (Onipe and Ramashia 2022). The polyphenols derived from Finger millet seed coat (FMSC) were analyzed for their antioxidant capacity against lipid oxidation in mayonnaise (Balasubramaniam et al. 2022). The study demonstrated that FMSC polyphenols (1.0 mg g^−1^) performed better than the synthetic antioxidant (BHT) in preventing oxidative rancidity of mayonnaise at 4℃ for seven weeks.

### Bioenergy production

Depletion of fossil fuels at alarming rates and emission of greenhouse gases in the atmosphere have necessitated the need to extensively explore sustainable resources for biofuel production. Lignocellulosic biomass residues are promising renewable resources for biofuel production (Kaur et al. 2023). A biorefinery approach was investigated (Jamaldheen et al. 2021) to meliorate Finger millet straw (FMS) an integrated production of bio-oil, bioethanol, and biochar. The FMS hydrolysate obtained after acid pretreatment of FMS was fermented using *Pichia stipites* NCIM-3497, after detoxification step, to produce ethanol (35%, w/w). The pretreated solid residue was pyrolyzed to yield bio-oil (42%, w/w) and biochar (27.2%, w/w). Further, the bio-oil obtained from FMS was processes to yield valuable compounds such as furfural, 4-allyl syringol and 1-(2-hydroxy-5-methylphenyl)-ethanone. The production of multiple value-added products from the Finger millet bioresource using a biorefinery concept helps to sustainably aim for circular economy from this underutilized millet crop. With an aim to leverage the energy potential of Finger millet residue, Hammerton et al. critically analysed its kinetic and thermodynamic parameters. The higher heating value of Finger millet residue was reported to be 15.46 MJ/kg, elucidating its feasibility for pyrolysis and energy generation (Hammerton et al. 2018). The FMS was found to have activation energy, enthalpy, entropy, Gibbs’s free energy, and frequency factor of 177.8 kJ mol^−1^, 174.17 kJ mol^−1^, − 5.56 J mol^−1^ K^−1^, 177.58 kJ mol^−1^, and 1.385 × 10^13^ s^−1^, respectively (Vikraman et al. 2021). These values highlight the viability of MS as a promising feedstock for pyrolysis and subsequent energy generation. Among various biomass to bioenergy methods, and pyrolysis is viewed as a cost-effective and eco-friendly technique that produces high-density biofuel. With minimal toxic by-product generation and flexibility with respect to all three forms of products (solid, liquid, and gas), this thermochemical process is a preferred waste management and energy recovery process from millet agro-residues (Tagade et al. 2021).

### Nutraceutical applications

The phenolic compounds, especially tannins, prevalent in the outer layers of Finger millet grain serve as a physical barrier to the fungal infestation (Devi et al. 2014). Due to the high polyphenol content, the Finger millet seed coat acidic methanol extract exhibited higher antibacterial and antifungal properties as compared to the whole flour extract. Due to the abundant presence of phenols and dietary fiber, the regular intake of Finger millet is known to reduce the risk of various gastrointestinal disorders as well as diabetes mellitus (Anitha et al. 2021). Streptozotocin-induced diabetic rats were fed with a diet containing 20% Finger millet seed coat matter (FMSCM) for six weeks that showed lesser degree of fasting hyperglycemia in comparison to the diabetic control. Further, the FMSCM-fed rats showed partial reversal of abnormalities in creatinine, urea, and serum albumin accompanied by notable reversal in hypertriacylglycerolaemia, hypercholesterolemia, neuropathy, and nephropathy (Shobana et al. 2010). The cyto-toxicity assay on HepG2 hepatic cancer cell lines highlighted the anti-cancer effects of Finger millet phenolics (Singh et al. 2015). The presence of phytochemicals such as alkaloids, terpenoids, phytocyanins, phytoestrogens, balsams, cardiac glycosides, tannins, steroids, etc., Finger millet acts as a prominent antioxidant, detoxifying agent and an immunity modulator (Saleh et al. 2013; Kumar et al. 2016). The calcium content of Finger millet seeds is reasonably higher (350 mg/100 g) than an average cow’s milk (112 mg/100 g milk). Considering the absence of lactose in this millet, it comes across an alternate easily digestible source of calcium for lactose-intolerant patients and weaning babies, (Wijesinha-Bettoni and Burlingame 2013), thereby posing as an affordable source for prevention of osteoporosis and other bone ailments (Kumar et al. 2016). It has also been reported that Finger millet possesses anti-ageing properties, since it can inhibit collagen cross-linking in the body, thereby reducing stiffness of elastic tissues in blood vessels, skin, tendons, etc. (Hegde et al. 2002). The nutritional significance of this millet crop must be efficiently translated into nutraceutical production to challenge the paradox of ailments and malnutrition.

The arabinoxylan-rich agricultural residues are currently being investigated for bioconversion of these materials into valuable macromolecules such as prebiotic xylooligosaccharides (XOS). These XOS are composed of xylobiose, xylotriose, and xylotetrose sugar molecules with a degree of polymerization ranging between 2 and 10 (Ravichandra et al. 2023). These non-digestible XOS have applications in food, feed formulations, medicine, and agriculture sector. The FMSC xylan-derived XOS were tested for their prebiotic efficacy. In comparison to the commercial XOS and dextrose, the FMSC XOS proved to be more effective substrate for inducing growth and cell mass of *Lactobacillus plantarum*, thereby validating its prebiotic potential (Palaniappan et al. 2017).

### Climate resilient potential of Finger millet

In view of climate change, abiotic and biotic stresses are major threats to the food security and crop productivity in the coming future and it is anticipated that by the year 2050 we need at least 70% more food production according to the reports of World food summit on food security. Plants develop several strategies to mitigate the effect of stress by adapting phenological, physiological, biochemical, and molecular changes. Finger millet being a minor millet, is a staple food crop for population living in Sub-Sahara Africa and Asia with high nutrition value and potential to grow in marginal agroecological zones where other crops find difficult to grow (Mbinda and Mukami 2021). Finger millet is one of minor millet that is adapted to grow under adverse condition and have high nutritional value, but it also suffers from various biotic (pest, pathogens) and abiotic (drought, salinity, heat and metal toxicity) stresses that have negative impact on plant growth, yield, and nutritional value too (Gull et al. 2014; Taranto et al. 2018; Singh Bakala et al. 2021; Ramesh et al. 2024).

### Resources available in Finger millet for crop improvement

Considering the importance of Finger millet in the changing scenario of climate change, shrinking cultivable land, increasing pressure to feed population, it became necessary to identify the genetic resource adaptable to various traits (Wambi et al. 2021). Currently, there are more than 34,675 Finger millet accessions that are available in various national and international gene banks worldwide in which India has the largest collection followed by Ethopia (Dwivedi et al. 2012). Limited studies have explored a core collection of Finger millet to identify accessions tolerant to drought, salinity, blast disease, and with high nutritional value. Among these, genotypes showing blast resistance include one wild type (*E. africana*) and four cultivated races of *E. coracana* (*vulgaris, plana, elongate, and compacta*) (Wambi et al. 2021). In term of genomic resources such as Expressed Sequence Tag (SSR) and Simple Sequence Repeat (SSR) and annotated genome there are not abundant resources as much as for other crop such as rice, maize, wheat, grasses. A study found that there were only 1934 EST for salinity, disease resistance and drought were available in the National Center for Biotechnology Information (NCBI) database which is quite less as compared to other crops (Antony Ceasar et al. 2018). Nevertheless, efforts are being made towards understanding the genomics of Finger millet, in recent years. Gimode et al. reported 10,337 SSR and 23,285 non-homologous SNP using Roche sequencing (Gimode et al. 2016). Further, a study (Pendergast et al. 2022) developed a high-density linkage map consisting 5422 markers. Currently in Finger millet three genomes are available in NCBI in which for cultivar KNE-796-S chromosome-level assembly and for PR202 and ML-365 scaffold-level assembly is available (https://www.ncbi.nlm.nih.gov/datasets/genome/?taxon=4511).

### Identification of Genes and QTLs associated with major stress tolerance in Finger millet

Currently, very limited literature is available on the quantitative trait loci (QTLs) identification in Finger millet, viz., Babu et al. (2014) identified QTL for blast by integration of GLM and MLM model which led to the identification of three common marker (RM262, FMBLEST32, and UGEP18) closely linked with blast resistance. On the basis of association mapping, a study identified two QTLs (*qLRDW.1*, *qLRDW.2*) associated with root dry weight under low phosphorus condition (Ramakrishna et al. 2018). Functional characterization of genes/Transcription Factors (TFs) associated with various biotic and abiotic stresses is one of the important aspects of the development of various stress-tolerant Finger millet. A study developed blast-resistant Finger millet using gene coding for antifungal protein of prawn that led to enhanced resistance (Latha et al. 2005). Mahalakshmi et al. (2006) reported the development of salt tolerant Finger millet using *P. coarctata* serine-rich-protein (*PcSrp*) encoding gene which increased the tolerance level of Finger millet up to 250 mM salt concentration. Later on, various reports come into light for the functional validation of genes/TFs either in the Finger millet or in the model system. For instance, *PcSrp* was overexpressed in Finger millet, resulting in increased salt tolerance up to 250 mM concentration (Mahalakshmi et al. 2006). Similarly, overexpression of *EcNAC1* in Tobacco showed a positive impact on plants during salinity and drought by accelerating root growth and recovery rate, and activating several other stress-responsive genes (Ramegowda et al. 2017). Overexpression of Rice Chitinase in Finger millet enhanced tolerance for blast diseases (Ignacimuthu and Ceasar 2012). The *mtlD* gene, when overexpressed in Finger millet, increased osmotic adjustment and chlorophyll retention under drought stress compared to wild-type plants (Hema et al. 2014). Overexpression of the *SbVPPase* gene from Sorghum bicolor in Finger millet enhanced salt tolerance by increasing antioxidant enzymes and chlorophyll content in transgenic lines (Anjaneyulu et al. 2014). *EcbZIP60*, highly expressed under drought, osmotic, salinity, and methyl viologen-induced stress, showed improved drought tolerance with high stomatal conductance and improved photosynthetic efficiency when overexpressed in Tobacco, although it led to growth retardation under normal conditions (Babitha et al. 2015). *EcDehydrin7* overexpression lines of Tobacco exhibited high expression and better tolerance to drought stress (Singh et al. 2015). Transgenic lines overexpressing *EcbHLH57* showed better tolerance to drought, salinity, and oxidative stress, with higher photosynthetic efficiency, stomatal conductance, and less accumulation of H_2_O_2_ and MDA levels (Babitha et al. 2015). *CIPK31*-like gene cloning and expression analysis demonstrated multiple abiotic stress tolerance, including drought, heat, salinity, and cold, by playing a role in signaling (Nagarjuna et al. 2016). *EcNAC 67* overexpression in Rice resulted in better tolerance to drought and salinity stress, increased relative water content (RWC), and decreased yield loss compared to wild-type plants under stress conditions (Rahman et al. 2016). Overexpression of *EcGBF3* in Arabidopsis thaliana improved tolerance to osmotic, salinity, and drought stress (Ramegowda et al. 2017). *EcbZIP17* overexpression in Tobacco enhanced abiotic stress tolerance, increased vegetative growth, and seed yield, and improved brassinosteroid signaling under normal conditions. Under stressful environments, it operated through the endoplasmic reticulum signaling pathway to improve tolerance (Ramakrishna et al. 2018). Finally, *EcDREB2A* overexpression in Tobacco enhanced heat tolerance by improving stomatal conductance, chlorophyll content, and maintaining ROS homeostasis (Singh et al. 2021).

Identification and validation of QTLs/genes relating to various biotic and abiotic stresses are key to the crop improvement. Finger millet crop improvement could be accelerated by integrating the cutting-edge technologies, such as RNA-sequencings, sRNA-sequencing, whole genome sequencing, proteomics, and metabolomics. With the availability of chromosome-level assembly of genome and regeneration protocol for agrobacterium-mediated transformation through tissue culture opens the door for crop improvement by gain-of-function and loss-of-function (CRISPR-cas9, RNAi, Prime editing, etc.) approach (Ignacimuthu and Ceasar 2012; Ngetich et al. 2018). Furthermore, we can employ speed breeding approach to accelerate the breeding programmes by developing efficient protocol of speed breeding. In the recent development synthetic biology gain a lot of attention where by utilizing the existing information we can design a completes organism by using computation, mathematical modelling, and functional characterization for stress tolerance (Mbinda and Mukami 2021).

## Conclusions

Finger millet is a promising crop with immense potential to address key challenges in food and nutrition security, as well as sustainable agriculture. Through its rich nutritional composition, diverse biological activities, and versatile industrial applications, Finger millet presents itself as a valuable resource for combating malnutrition and promoting human health. Furthermore, its remarkable climate resilience makes it a vital component of climate-smart agriculture, offering resilience against environmental stresses and contributing to agricultural sustainability. Looking forward, Finger millet holds significant future potential in several domains. First, its role in addressing malnutrition and enhancing food security is becoming even more crucial, particularly in regions vulnerable to food insecurity and climate change impacts. Second, the industrial applications of Finger millet, including its use in nutraceuticals, functional foods, and bioenergy production, are likely to expand, driven by increasing consumer demand for healthy and sustainable products. Additionally, advancements in crop improvement techniques, utilizing genetic resources and stress tolerance traits, offer opportunities for developing high-yielding and resilient Finger millet cultivars tailored to diverse agroecological conditions. Moreover, the designation of the International Year of Millets in 2023 highlights the global recognition of the nutritional significance and agricultural potential of millets, including Finger millet. This initiative not only raises awareness about the nutritional benefits of millets but also encourages investment in research, development, and promotion of millet-based agriculture and food systems. In summary, Finger millet represents a valuable asset in the goal for sustainable and resilient food systems. By utilizing its nutritional, biological, and industrial potential, coupled with strategic investments and policy support, Finger millet can emerge as a cornerstone of global efforts towards achieving a more food-secure, healthy, and sustainable future.

## Data Availability

Not applicable.
