# Correction: Finger millet (*Eleusine coracana* L.): from staple to superfood—a comprehensive review on nutritional, bioactive, industrial, and climate resilience potential

**DOI:** 10.1007/s00425-024-04516-w

**Published:** 2024-08-28

**Authors:** Simardeep Kaur, Arti Kumari, Karishma Seem, Gurkanwal Kaur, Deepesh Kumar, Surbhi Verma, Naseeb Singh, Amit Kumar, Manish Kumar, Sandeep Jaiswal, Rakesh Bhardwaj, Binay Kumar Singh, Amritbir Riar

**Affiliations:** 1 ICAR-Research Complex for North Eastern Hill Region, Umiam, Meghalaya 793103 India; 2 Bihar Agricultural University, Sabour, Bhagalpur, 813210 India; 3 ICAR-Indian Agricultural Research Institute, New Delhi, 110012 India; 4 Punjab Agricultural University, Ludhiana, Punjab 141004 India; 5 ICAR-National Institute of Plant Biotechnology, New Delhi, 110012 India; 6 College of Agriculture and Applied Sciences, Utah State University, Logan, UT 84322 USA; 7 ICAR-National Bureau of Plant Genetic Resources, New Delhi, 110012 India; 8 Department of International Cooperation, Research Institute of Organic Agriculture, FiBL, 11 Frick, Switzerland

**Correction: Planta** 10.1007/s00425-024-04502-2

In this article the affiliation details for Manish Kumar were incorrectly given as ‘Bihar Agricultural University, Sabour, Bhagalpur 813210, India’ but should have been ‘ICAR-IARI, New Delhi, India’.

